# Non-inferiority study of the efficacy of two hyaluronic acid products in post-extraction sockets of impacted third molars

**DOI:** 10.1186/s40902-020-00287-y

**Published:** 2020-12-09

**Authors:** Hyunwoo Yang, Junghun Kim, Jihong Kim, Dongwook Kim, Hyung Jun Kim

**Affiliations:** Department of Oral & Maxillofacial Surgery, Yonsei University College of Dentistry, 50-1 Yonsei-ro, Seodaemun-gu, Seoul, 03722 Republic of Korea

**Keywords:** Mucobarrier, Aloclair, Hyaluronic acid, Double blind test, Mandibular third molar

## Abstract

**Background:**

Hyaluronic acid (HA) is well known to exert an anti-inflammatory effect during oral wound healing and is commonly applied after tooth extraction. However, no double-blind randomized controlled study comparing two hyaluronate mouthwash products has been conducted so far. The aim of this study was to comparatively analyze the efficacy of Mucobarrier® and Aloclair® in terms of clinical symptoms.

**Results:**

A total of 112 patients were randomly assigned to assess the degree of discomfort, pain reduction, redness, burning sensation, and swelling between two groups on the day of surgery and 7 days later in a double blind test, with a total 56 Aloclair patients and 56 Mucobarrier patients. There was no statistically significant difference in the overall discomfort, degree of pain reduction, redness, burning sensation, and swelling between the Mucobarrier and Aloclair groups.

**Conclusion:**

The local application of hyaluronic acid mouth wash after wisdom tooth extraction is beneficial in reducing overall discomfort and pain reduction, and the clinical utility of Mucobarrier® is no different from Aloclair®.

**Trial registration:**

Institutional Review Board of Yonsei University College of Dentistry, 2-2018-0036. Registered 10 September 2018—prospectively registered, https://eirb.yuhs.ac/

## Background

The surgical extraction of wisdom teeth is one of the most common procedures in oral surgery. Numerous complications and sequelae can develop which negatively affect patients’ quality of life such as pain, swelling, bleeding, laceration of gingiva or oral mucosa, and trismus [1, 2]. Steroid agents, as well as the application of hyaluronic acid, are known to be effective in minimizing these discomforts [2].

Hyaluronic acid (HA) is a polysaccharide of the extracellular matrix found in various body tissues including connective tissue, epithelium, and nerve tissues. HA can be used safely in medicine because it is nonimmunogenic and nontoxic [3]. It plays an important role in wound healing through accumulation on the wound area, reinforcing angiogenesis and suppressing the growth of fibroblasts by absorbing free radicals, thereby reducing scar formation [4]. HA also contributes to maintaining the constancy of the epidermis by maintaining osmotic pressure; in addition, its many charged branches can reduce pain by retaining a large amount of moisture [5]. Previous studies of patients with recurrent apnea stomatitis found that the pain in the group with hyaluronic acid was significantly reduced [6, 7]. A study of pain control after CO_2_ laser surgery also showed reduced pain in the group to which hyaluronic acid was applied [8]. Hyaluronic acids are also used in the dental field in the form of gargle solution and are known to be particularly effective in wound healing and pain control.

The purpose of this clinical trial is to investigate whether there is any benefit in local application of HA solution (Mucobarrier® and Aloclair®) in wound healing and pain control after extraction of wisdom teeth and to prove the clinical effectiveness of Mucobarrier® is not inferior to Aloclair® through a non-inferiority test. To the best of our knowledge, this study comprises largest cohort group of patients who went under impacted third molar extraction and also is the first study which executed double-blind randomized control trial.

## Methods

### Patients

Patients who visited the Department of Oral and Maxillofacial Surgery of Yonsei University Dental Hospital from January 2019 to January 2020 were investigated. This study was reviewed and approved by the Institutional Review Board of Yonsei University College of Dentistry (approval number 2-2018-0036).

The initial number of patients was 116, a 10% dropout rate resulting in a total of 104 people, including 52 of the Aloclair® group and 52 of the Mucobarrier® group. Adults aged 19 or older who required surgical extraction of wisdom tooth, who had voluntarily decided to participate in this clinical trial, and who could participate for the entire pre-clinical period were chosen as study participants. Pregnant or breastfeeding women and those who did not understand the research were excluded from the study.

**Fig. 1:**
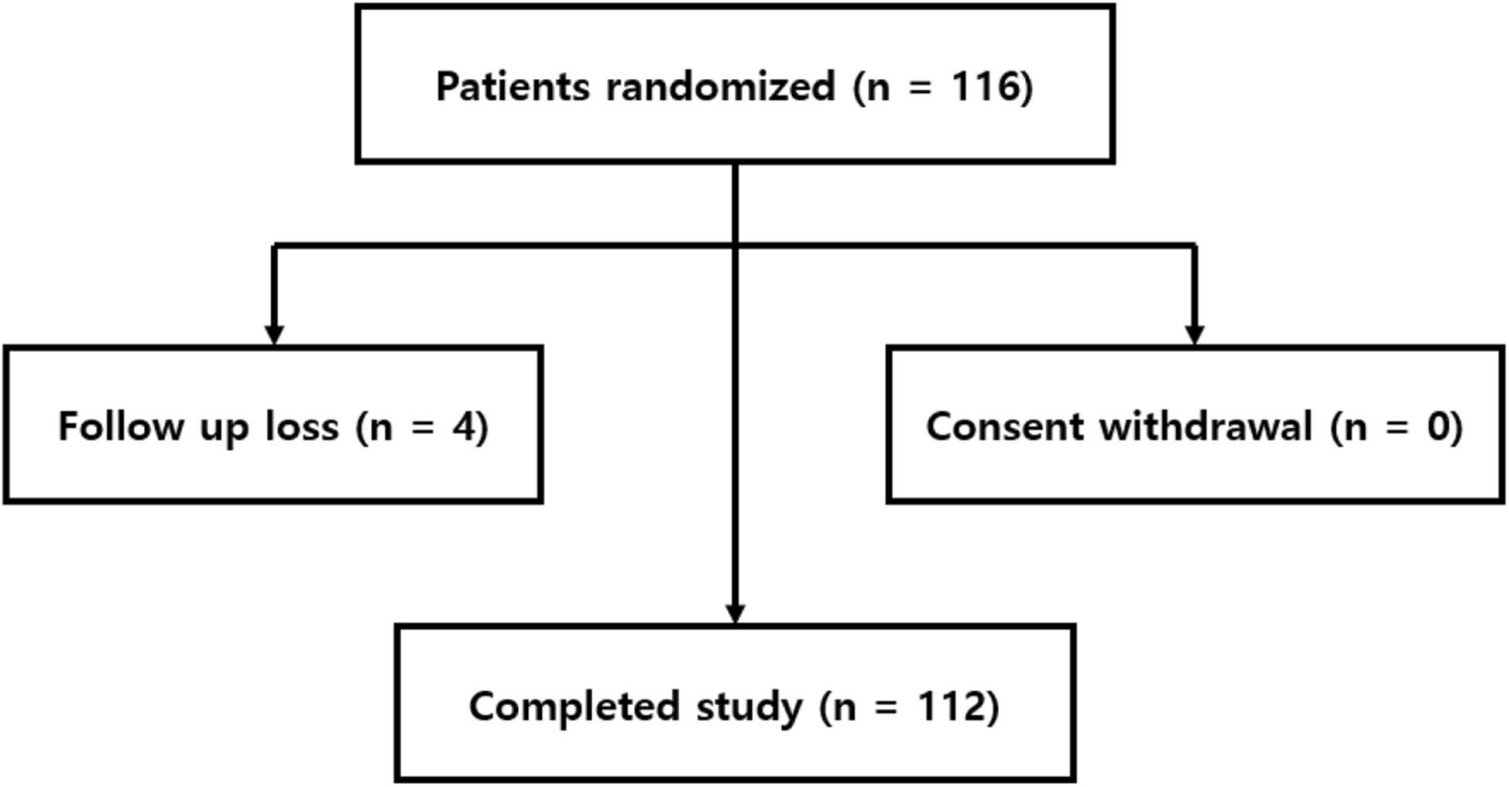
Patient disposition

Of the 116 recruited participants, four were excluded due to follow-up loss. The final number of participants was 112, 56 assigned to the Aloclair® group and 56 to the Mucobarrier® group (Fig. 1).

### Materials

**Fig. 2:**
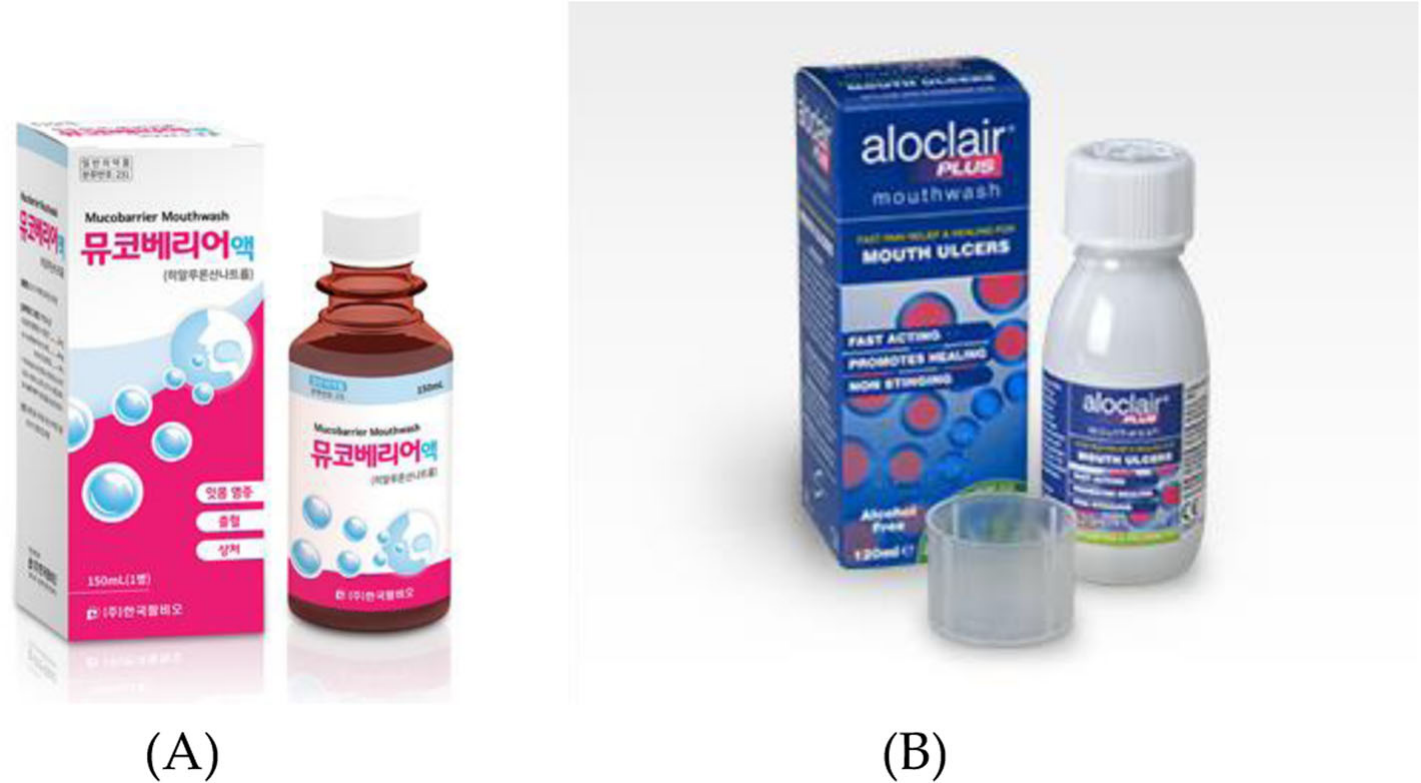
Hyaluronic acid products. **a** Mucobarrier®. **b** Aloclair®

Mucobarrier® and Aloclair® are oral prophylactic agents containing hyaluronic acid and are made up of similar ingredients. To prevent the test subjects from distinguishing the products, no product name was visible on the container, nor was the agent visible inside (Fig. 2).

### Patient assignment

The order of assignment was decided based on a randomization of the registration numbers of the test subjects performed using a computerized random allocation program; neither the test subjects nor experimenters were able to know which product had been applied.

### Patient evaluation

The degree of pain, burning sensation, redness, and swelling on the day of the extraction was evaluated.

### Data analysis and statistics

As this study is prospective, we evaluated differences in the therapeutic effects between the two groups by dividing the total 112 patients into 56 Aloclair® group and 56 Mucobarrier® group in a double-blind randomized control trial. The statistical analysis was done using R version 3.6.3 (R Foundation, Vienna, Austria).

## Results

This prospective study compared the therapeutic effects of two HA products on individuals assigned to test and control groups. Among a total 112 people (45 males and 67 females), 56 were assigned to the Aloclair® group and 56 to the Mucobarrier® group. Ages ranged from 18 to 71, with median age 29.5 and average age 32.0 (Table 1).

**Table 1:** Demographic factors of participants

Factor	Aloclair (***N*** = 56)	Mucobarrier (***N*** = 56)	***N*** (percentage)
**Age**			
**Young adult (18–30 years)**	28	29	57 (50.9%)
**Middle adult (31–50 years)**	20	23	43 (39.1%)
**Young adult (> 56 years)**	7	3	10 (9.1%)
**Gender**			
**Male**	25	19	44 (40.0%)
**Female**	30	36	66 (60.0%)

Overall discomfort, pain relief, burning sensation, redness, and swelling were evaluated immediately after surgery and 1 week after surgery. A visual analog scale was used to assess the amount of pain, and there were no statistically significant differences in overall discomfort, pain relief, burning sensation, or swelling between the two groups (Tables 2 and 3).

**Table 2:** Post-operative discomfort and pain

Group	Aloclair (*N* = 56)	Mucobarrier (*N* = 56)	*p*
Subjective_overall_discomfort			0.538
1. Very good	9 (15.1%)	14 (23.6%)	
2. Comfortable	35 (64.2%)	35 (63.6%)	
3. Slight discomfort	11 (18.9%)	6 (10.9%)	
4. Very uncomfortable	1 (1.9%)	1 (1.8%)	
Pain reduction	5.0 [2.0;6.0]	5.0 [3.0;5.5]	0.804

“Minimal” refers to a noticeable state, but not apparent compared to the contralateral side

**Table 3:** Post-operative burning sensation, redness, and swelling

Group	Aloclair (*N* = 56)	Mucobarrier (*N* = 56)	*p*
Burning sensation			1
1. Absent	49 (88.7%)	48 (87.3%)	
2. Present	7 (11.3%)	8 (12.7%)	
Redness			0.681
1. Absent	26 (45.3%)	27 (49.1%)	
2. Minimal	20 (35.8%)	22 (38.2%)	
3. Apparent	10 (18.9%)	7 (12.7%)	
Swelling			0.936
1. Absent	23 (39.6%)	23 (40.0%)	
2. Minimal	25 (45.3%)	26 (47.3%)	
3. Apparent	8 (15.1%)	7 (12.7%)	

“Minimal” refers to a noticeable state, but not apparent compared to the contralateral side

## Discussion

After extraction of the wisdom tooth, complications such as pain, redness, swelling, and burning sensation always accompany though in varying degrees. Many studies have attempted to minimize these, and hyaluronic acid has been reported to have a positive effect on surgical wound healing as well as on intraoral wound healing and pain relief [9, 10]. Nolan et al. [7] said that HA can reduce pain in the wound through barrier formation. HA itself has a very high osmotic pressure, which allows it to maintain sufficient moisture around damaged tissue during inflammatory reaction, thus stabilizing the wound and helping cell migration and proliferation.

A previous study on the treatment of oral mucosa with Aloclair after chemotherapy or radiation therapy found that it soothes irritated tissue and reduces pain by forming a protective membrane over oral mucosa. The main components of Aloclair are purified water, maltodextrin, propylene glycol, polyvinyl pyrrolidone (a hydrophilic polymer which hydrates tissue, thus accelerating wound healing in animal models and human wound), sodium hyaluronic acid (which occurs naturally in body, forms mucous, and thus hydrates mucous membranes while acting as coating material on membranes), and glycyrrhetinic acid (licorice extract, a cyclo-oxygenase inhibitor that mediates healing through antibacterial properties) [11].

The main ingredients of Mucobarrier are sodium hyaluronic acid and a trace amount of aloe, which is known to have anti-inflammatory and antioxidant properties.

Visualized analog scale (VAS), a simple method of self-assessment of subjective sensations, including pain, was used to measure the degree of pain in this study [12]. It is widely regarded as a valid method of quantitatively measuring pain and has been used to study pain and inflammation after extraction of the third molar and to measure pain of patients after oral mucosa biopsy [13, 14]. Although many of the variables such as threshold for pain, mental status on the day of operation, and emotional state constitute subjective criteria and thus limitations of the study, we interpreted the pain relief effect of hyaluronic acid as valid because the only ingredient in common between Mucobarrier and Aloclair is hyaluronic acid and there was definite pain reduction in both groups.

This study found no statistically significant difference between Mucobarrier and Aloclair in terms of general discomfort or pain relief nor any statistically significant difference in local heat, redness, or swelling.

Reasons for the lack of significant differences between the two groups are as follows. First, this study compared the clinical symptoms on the day of extraction and a week later, an interval which is too long considering that clinical symptoms of postoperative inflammatory reactions reach their peak after 1 or 2 days and generally subside within a week. Thus, a week after surgery is important when considering the relevant factors affecting the early stages of wound healing [15]. However, oral mucosa receives a more abundant blood supply compared to other parts of the body and wounds heal faster than in other parts of the body. As primary healing occurs within 2 weeks in most cases, the 1-week interval of evaluation in this study could not fully reflect the entire healing process. This may have contributed to our outcome. Nolan et al. applied HA on aphthous stomatitis ulcerative lesion and followed up every day to observe the differences between the HA and placebo group. There were significant differences on day 4, suggesting that significant differences might be seen during the first week after surgery.

Furthermore, like previous studies including HA, this study was based on limited sample sizes and designed without negative control. Although previous study about discomfort after third molar surgery without any other agents applied showed higher percentage of discomfort compared to the result of our study with HA applied, additional studies of different design and with more participants including negative control would yield more reliable contrastive research [16].

## Conclusions

Based on this study, the local application of hyaluronic acid solution after extraction of wisdom tooth is beneficial for post-operative discomfort and pain reduction, and the clinical effect of Mucobarrier® is not inferior to Aloclair®.

## Data Availability

All data analyzed during this study are included in this published article.

## Abbreviations

HA: Hyaluronic acid
VAS: Visualized analog scale

## References

[1] Cervino G, Cicciù M, Biondi A, Bocchieri S, Herford AS, Laino L (2019). Antibiotic prophylaxis on third molar extraction: systematic review of recent data. Antibiotics.

[2] Van Der Goes MC, Jacobs JW, Bijlsma JW (2014). The value of glucocorticoid co-therapy in different rheumatic diseases-positive and adverse effects. Arthritis Res Ther.

[3] Sahayata VN, Bhavsar NV, Brahmbhatt NA (2014). An evaluation of 0.2% hyaluronic acid gel (Gengigel®) in the treatment of gingivitis: a clinical & microbiological study. Oral Health Dent Manage.

[4] Foschi D, Castoldi L, Radaelli E, Abelli P, Calderini G, Rastrelli A (1990). Hyaluronic acid prevents oxygen free-radical damage to granulation tissue: a study in rats. Int J Tissue React.

[5] Voinchet V, Vasseur P, Kern J (2006). Efficacy and safety of hyaluronic acid in the management of acute wounds. Am J Clin Dermatol.

[6] Casale M, Moffa A, Vella P, Sabatino L, Capuano F, Salvinelli B (2016). Hyaluronic acid: perspectives in dentistry. A systematic review. Int J Immunopathol Pharmacol.

[7] Nolan A, Baillie C, Badminton J, Rudralingham M, Seymour R (2006). The efficacy of topical hyaluronic acid in the management of recurrent aphthous ulceration. J Oral Pathol Med.

[8] Origoni M, Cimmino C, Carminati G, Iachini E, Stefani C, Girardelli S (2016). Postmenopausal vulvovaginal atrophy (VVA) is positively improved by topical hyaluronic acid application. A prospective, observational study. Eur Rev Med Pharmacol Sci.

[9] Gocmen G, Aktop S, Tüzüner B, Goker B, Yarat A (2017). Effects of hyaluronic acid on bleeding following third molar extraction. J Appl Oral Sci.

[10] Gocmen G, Gonul O, Oktay NS, Yarat A, Goker K (2015). The antioxidant and anti-inflammatory efficiency of hyaluronic acid after third molar extraction. J Cranio-Maxillofac Surg.

[11] Innocenti M, Moscatelli G, Lopez S (2002). Efficacy of gelclair in reducing pain in palliative care patients with oral lesions: preliminary findings from an open pilot study. J Pain Symptom Manag.

[12] Seymour RA, Meechan JG, Blair GS (1985). An investigation into post-operative pain after third molar surgery under local analgesia. Br J Oral Maxillofac Surg.

[13] Penarrocha M, Garcia B, Marti E, Balaguer J (2006). Pain and inflammation after periapical surgery in 60 patients. J Oral Maxillofac Surg.

[14] Van Buren J, Kleinknecht RA (1979). An evaluation of the McGill Pain Questionnaire for use in dental pain assessment. Pain.

[15] Sato FRL, Asprino L, De Araújo DES, De Moraes M (2009). Short-term outcome of postoperative patient recovery perception after surgical removal of third molars. J Oral Maxillofac Surg.

[16] Lopes V, Mumenya R, Feinmann C, Harris M (1995). Third molar surgery: an audit of the indications for surgery, post-operative complaints and patient satisfaction. Br J Oral Maxillofac Surg.

